# Formamidinium iodide: crystal structure and phase transitions

**DOI:** 10.1107/S205698901700425X

**Published:** 2017-03-24

**Authors:** Andrey A. Petrov, Eugene A. Goodilin, Alexey B. Tarasov, Vladimir A. Lazarenko, Pavel V. Dorovatovskii, Victor N. Khrustalev

**Affiliations:** a Department of Materials Science, Lomonosov Moscow State University, Lenin Hills, 119991 Moscow, Russian Federation; b Department of Chemistry, Lomonosov Moscow State University, Lenin Hills, 119991 Moscow, Russian Federation; c National Research Centre ‘Kurchatov Institute’, 1 Acad. Kurchatov Sq., Moscow 123182, Russian Federation; d Inorganic Chemistry Department, Peoples’ Friendship University of Russia (RUDN University), 6 Miklukho-Maklay St., Moscow 117198, Russian Federation; e X-Ray Structural Centre, A. N. Nesmeyanov Institute of Organoelement Compounds, Russian Academy of Sciences, 28 Vavilov St., B-334, Moscow 119991, Russian Federation

**Keywords:** crystal structure, formamidinium iodide, phase transitions, powder diffraction, synchrotron radiation

## Abstract

At a temperature of 100 K, CH_5_N_2_^+^·I^−^ (**I**), crystallizes in the monoclinic space group *P*2_1_/*c*. The formamidinium cation adopts a planar symmetrical structure [the r.m.s. deviation is 0.002 Å, and the C—N bond lengths are 1.301 (7) and 1.309 (8) Å]. The iodide anion does not lie within the cation plane, but deviates from it by 0.643 (10) Å. The cation and anion of **I** form a tight ionic pair by a strong N—H⋯I hydrogen bond. In the crystal of **I**, the tight ionic pairs form hydrogen-bonded zigzag-like chains propagating toward [20-1] *via* strong N—H⋯I hydrogen bonds. The hydrogen-bonded chains are further packed in stacks along [100]. The thermal behaviour of **I** was studied by different physicochemical methods (thermogravimetry, differential scanning calorimetry and powder diffraction). Differential scanning calorimetry revealed three narrow endothermic peaks at 346, 387 and 525 K, and one broad endothermic peak at ∼605 K. The first and second peaks are related to solid–solid phase transitions, while the third and fourth peaks are attributed to the melting and decomposition of **I**. The enthalpies of the phase transitions at 346 and 387 K are estimated as 2.60 and 2.75 kJ mol^−1^, respectively. The X-ray powder diffraction data collected at different temperatures indicate the existence of **I** as the monoclinic (100–346 K), ortho­rhom­bic (346–387 K) and cubic (387–525 K) polymorphic modifications.

## Abstract 2

Crystal structure, thermal behaviour and phase transitions of formamidinium iodide were studied by DTG, DSC, powder diffraction and X-ray crystallography.

## Chemical context   

Compounds with the general formula *ABX*_3_ [where *A* denotes an organic cation *e.g.* methylammonium (MA, CH_3_NH_3_^+^) or formamidinium [FA = CH(NH_2_)_2_, CH_3_NH_3_]; *B* = Pb, Sn; *X* = I, Br, Cl] belong to a class of hybrid organic–inorganic perovskites and perform as outstanding light harvesters. These compounds gave birth to a new field of photovoltaics – perovskite solar cells – when Kojima and co-authors used (MA)PbI_3_ as a light sensitizer for the first time in dye-sensitized solar cells (DSSCs) in 2009 and showed 3.8% efficiency (Kojima *et al.*, 2009 ▸). Since then, a revolutionary breakthrough has occured in this area and the highest efficiency now has reached 22.1%.

In 2014, the formamidinium cation was proposed to replace methyl­ammonium and the further investigation of (FA)PbI_3_ disclosed its superiority to (MA)PbI_3_ (Koh *et al.*, 2014 ▸; Pang *et al.*, 2014 ▸). In particular, it was found that (FA)PbI_3_ exhibits higher thermal and moisture stability and has a lower bandgap than (MA)PbI_3_ which gives a greater capacity for sunlight absorption (Koh *et al.*, 2014 ▸; Han *et al.*, 2016 ▸). Recently, it was shown that the properties of the compounds may be further optimized by tuning the MA/FA ratio and an efficiency of 20.5% has been reached for a mixed compound (Li *et al.*, 2016 ▸; Jeon *et al.*, 2015 ▸).

The main precursors to obtain (FA)PbI_3_ are PbI_2_ and formamidinium iodide (FA)I. Several methods of perovskite synthesis include steps where it can be obtained directly from (FA)I in a crystalline form (Zhou *et al.*, 2015 ▸; Leyden *et al.*, 2015 ▸). It also appears in a crystalline form and leads to a formation of low-dimensional phases when an excess of it is taken (Xi *et al.*, 2016 ▸; Ma *et al.*, 2017 ▸). Thus, the understanding of the (FA)I crystal structure gives valuable information for understanding the crystallization of formamidinium-based lead halide perovskites. Knowledge of the (FA)I crystal structure may also be of particular inter­est for computer simulations of the processes related to the crystallization of these perovskites. Surprisingly, despite the hundreds of papers published over the last several years that have mentioned (FA)I as a major precursor for hybrid lead halide perovskites, its crystal structure has remained unknown so far.
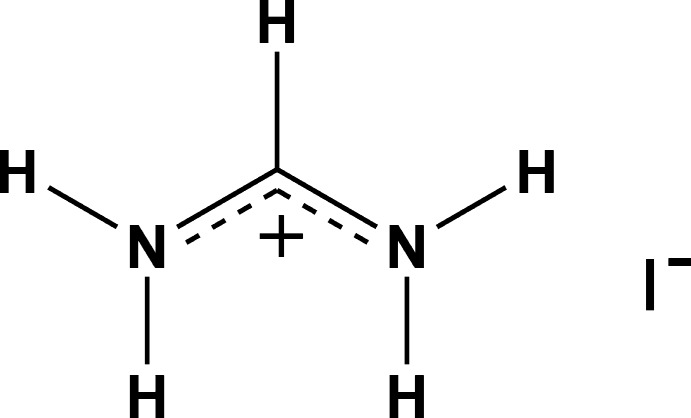


In this work, we investigated the structure of (FA)I (**I**) and its thermal behaviour by different physico-chemical methods.

## Structural commentary   

**Figure 1:**
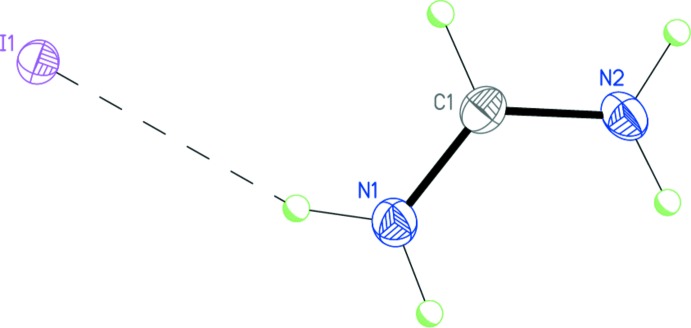
The mol­ecular structure of salt **I**. Displacement ellipsoids are shown at the 50% probability level. H atoms are presented as small spheres of arbitrary radius. Dashed line indicates the inter­molecular N—H⋯I hydrogen bond.

At a temperature of 100 K, compound **I** crystallizes in the monoclinic space group *P*2_1_/*c*. The formamidinium cation adopts a planar symmetrical structure [r.m.s. deviation is 0.002 Å, and the C—N bond lengths are 1.301 (7) and 1.309 (8) Å; Fig. 1 ▸]. The iodide anion does not lie within the cation plane, but deviates from it by 0.643 (10) Å. The cation and anion in **I** form a tight ionic pair by the strong N1—H1*A*⋯I1 hydrogen bond (Table 1 ▸ and Fig. 1 ▸).

**Figure 2:**
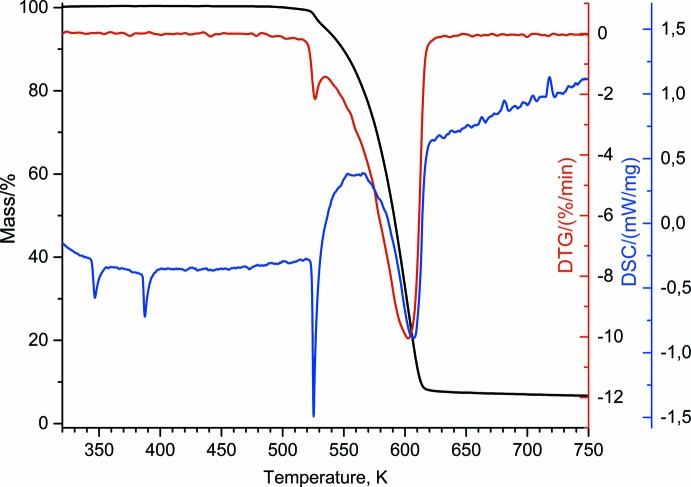
Thermogravimetry and differential scanning calorimetry analyses for **I**.

In order to understand the thermal behaviour of **I** at elevated temperatures, the sample was investigated by TG and DSC methods in the temperature region from 293 to 750 K at a rate of 5 K min^−1^. The mass loss started from ∼520 K (Fig. 2 ▸). Differential scanning calorimetry revealed three narrow endothermic peaks at 346, 387 and 525 K, and one broad endothermic peak at ∼605 K. The first and the second peaks are related to solid–solid phase transitions, while the third and the fourth peaks are attributed to the melting and decomposition of **I**. Enthalpy of the phase transitions at 346 and 387 K are estimated as 2.24 and 2.87 kJ mol^−1^, respectively.

**Figure 3:**
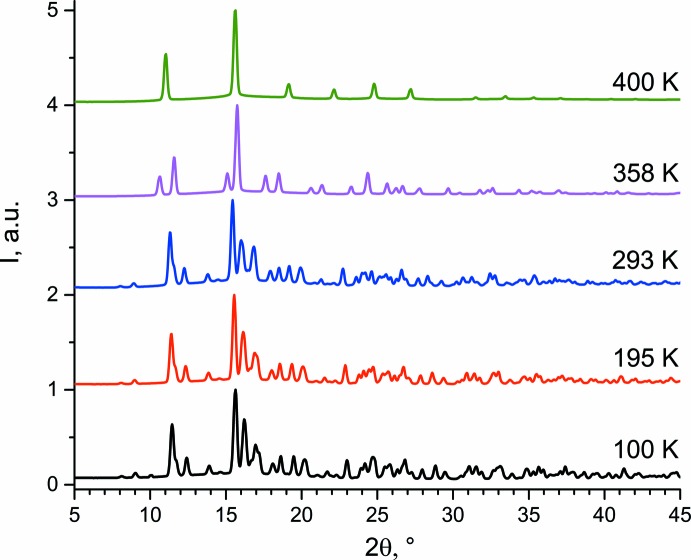
X-ray powder diffraction data for **I** at different temperatures.

The X-ray powder diffraction data collected at different temperatures confirm the existence of different phases (Fig. 3 ▸). At low temperatures, salt **I** exists in a monoclinic phase and exhibits a significant change of the parameters with a rise in temperature (100 → 195 → 293 K, Fig. 3 ▸). A phase existing at 358 K is indexed in an ortho­rhom­bic crystal system [*a* = 7.3915 (8) Å, *b* = 6.3358 (8) Å, *c* = 5.2391 (9) Å; *M*(20) = 25, *F*(20) = 45]. Another high-temperature phase is cubic, exhibiting only a few reflections at 400 K [*a* = 5.0571 (5) Å; *M*(13) = 126, *F*(13) = 109]. It seems to be a plastic phase similar to a plastic phase for methyl­ammonium iodide (Ishida *et al.*, 1995 ▸; Yamamuro *et al.*, 1992 ▸).

## Supra­molecular features   

**Figure 4:**
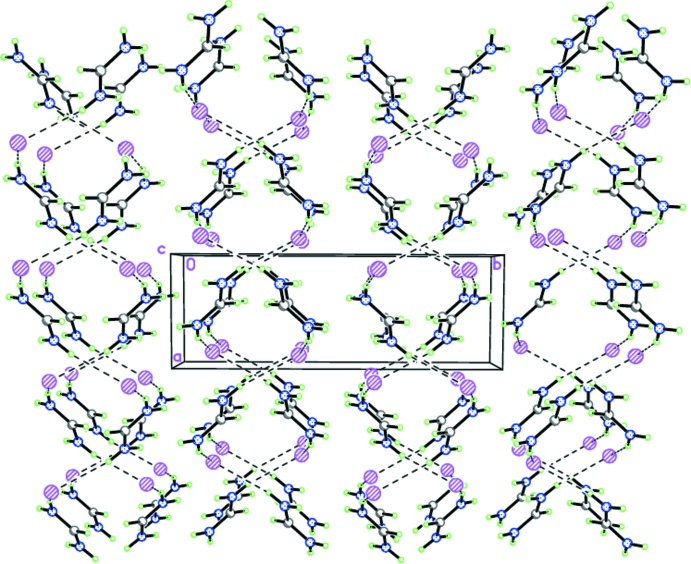
The crystal structure of **I** demonstrating the hydrogen-bonded zigzag-like chains propagating toward [20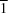]. Dashed lines indicate the inter­molecular N—H⋯I hydrogen bonds.

In the crystal of **I**, the tight ionic pairs form hydrogen-bonded zigzag-like chains propagating toward [20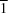] by the strong inter­molecular N2—H2*A*⋯I1^i^ hydrogen bonds (Table 1 ▸ and Fig. 4 ▸). The hydrogen-bonded chains are further packed in stacks along [100] (Fig. 4 ▸) [symmetry code: (i) *x* − 1, −*y* + 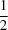, *z* + 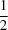].

## Synthesis and crystallization   

Polycrystalline powder of **I** was purchased from Dyesol and used without further purification. Single crystals suitable for X-ray structural study were obtained by recrystallization from an anhydrous ethanol solution by slow cooling.

## Refinement   

Crystal data, data collection and structure refinement details are summarized in Table 2 ▸. X-ray diffraction study of **I** was carried out on the ‘Belok’ beamline of the National Research Center ‘Kurchatov Institute’ (Moscow, Russian Federation) using a Rayonix SX165 CCD detector. Reflection intensities measured were corrected for absorption using the *Scala* (Evans, 2006 ▸) program.

The H atoms of the NH_2_ groups were localized in the difference Fourier map and refined with fixed positional and isotropic displacement parameters [*U*_iso_(H) = 1.2*U*_eq_(N)]. The CH hydrogen was placed in a calculated position, with C—H = 0.95 Å, and refined in the riding model with a fixed isotropic displacement parameter [*U*_iso_(H) = 1.2*U*_eq_(C)].

## Supplementary Material

Crystal structure: contains datablock(s) global, I. DOI: 10.1107/S205698901700425X/lh5840sup1.cif

Structure factors: contains datablock(s) I. DOI: 10.1107/S205698901700425X/lh5840Isup2.hkl

Supporting information file. DOI: 10.1107/S205698901700425X/lh5840Isup3.cml

CCDC reference: 1538402

Additional supporting information:  crystallographic information; 3D view; checkCIF report

## Figures and Tables

**Table 1:** Hydrogen-bond geometry (Å, °)

*D*—H⋯*A*	*D*—H	H⋯*A*	*D*⋯*A*	*D*—H⋯*A*
N1—H1*A*⋯I1	0.90	2.77	3.612 (5)	156
N2—H2*A*⋯I1^i^	0.90	2.74	3.622 (4)	166

Symmetry code: (i) 
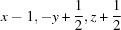
.

**Table 2:** Experimental details

Crystal data	
Chemical formula	CH_5_N_2_^+^·I^−^
*M*_r_	171.97
Crystal system, space group	Monoclinic, *P*2_1_/*c*
Temperature (K)	100
*a*, *b*, *c* (Å)	4.8211 (6), 13.776 (3), 7.0113 (10)
β (°)	98.06 (3)
*V* (Å^3^)	461.06 (14)
*Z*	4
Radiation type	Synchrotron, λ = 0.96990 Å
μ (mm^−1^)	15.38
Crystal size (mm)	0.06 × 0.05 × 0.03

Data collection	
Diffractometer	Rayonix SX165 CCD
Absorption correction	Multi-scan (*SCALA*; Evans, 2006 ▸)
*T*_min_, *T*_max_	0.400, 0.600
No. of measured, independent and observed [*I* > 2σ(*I*)] reflections	5111, 949, 894
*R*_int_	0.070
(sin θ/λ)_max_ (Å^−1^)	0.642

Refinement	
*R*[*F* ^2^ > 2σ(*F* ^2^)], *wR*(*F* ^2^), *S*	0.039, 0.093, 1.06
No. of reflections	949
No. of parameters	38
H-atom treatment	H-atom parameters constrained
Δρ_max_, Δρ_min_ (e Å^−3^)	0.87, −0.91

Computer programs: *Marccd* (Doyle, 2011 ▸), *iMosflm* (Battye *et al.*, 2011 ▸), *SHELXT* (Sheldrick, 2015*a* ▸), *SHELXL2014* (Sheldrick, 2015*b* ▸) and *SHELXTL* (Sheldrick, 2008 ▸).

## supplementary crystallographic information

### Crystal data

**Table:**

CH_5_N_2_^+^·I^−^	*F*(000) = 312
*M**_r_* = 171.97	*D*_x_ = 2.477 Mg m^−^^3^
Monoclinic, *P*2_1_/*c*	Synchrotron radiation, λ = 0.96990 Å
*a* = 4.8211 (6) Å	Cell parameters from 600 reflections
*b* = 13.776 (3) Å	θ = 4.0–36.0°
*c* = 7.0113 (10) Å	µ = 15.38 mm^−^^1^
β = 98.06 (3)°	*T* = 100 K
*V* = 461.06 (14) Å^3^	Prism, colourless
*Z* = 4	0.06 × 0.05 × 0.03 mm

### Data collection

**Table:**

Rayonix SX165 CCD diffractometer	894 reflections with *I* > 2σ(*I*)
φ scan	*R*_int_ = 0.070
Absorption correction: multi-scan (Scala; Evans, 2006)	θ_max_ = 38.5°, θ_min_ = 4.0°
*T*_min_ = 0.400, *T*_max_ = 0.600	*h* = −6→6
5111 measured reflections	*k* = −17→17
949 independent reflections	*l* = −8→7

### Refinement

**Table:**

Refinement on *F*^2^	Secondary atom site location: difference Fourier map
Least-squares matrix: full	Hydrogen site location: mixed
*R*[*F*^2^ > 2σ(*F*^2^)] = 0.039	H-atom parameters constrained
*wR*(*F*^2^) = 0.093	*w* = 1/[σ^2^(*F*_o_^2^) + 0.7865*P*] where *P* = (*F*_o_^2^ + 2*F*_c_^2^)/3
*S* = 1.06	(Δ/σ)_max_ < 0.001
949 reflections	Δρ_max_ = 0.87 e Å^−^^3^
38 parameters	Δρ_min_ = −0.91 e Å^−^^3^
0 restraints	Extinction correction: SHELXL2014 (Sheldrick, 2015b), Fc^*^=kFc[1+0.001xFc^2^λ^3^/sin(2θ)]^-1/4^
Primary atom site location: difference Fourier map	Extinction coefficient: 0.0064 (11)

### Special details

**Table:**

Geometry. All esds (except the esd in the dihedral angle between two l.s. planes) are estimated using the full covariance matrix. The cell esds are taken into account individually in the estimation of esds in distances, angles and torsion angles; correlations between esds in cell parameters are only used when they are defined by crystal symmetry. An approximate (isotropic) treatment of cell esds is used for estimating esds involving l.s. planes.

### Fractional atomic coordinates and isotropic or equivalent isotropic displacement parameters (Å^2^)

**Table:**

	*x*	*y*	*z*	*U*_iso_*/*U*_eq_	
I1	0.86658 (6)	0.38577 (2)	0.20022 (5)	0.0218 (2)	
N1	0.6120 (9)	0.4171 (3)	0.6569 (7)	0.0250 (10)	
H1A	0.7250	0.4174	0.5650	0.030*	
H1B	0.6251	0.4648	0.7454	0.030*	
N2	0.2540 (9)	0.3389 (3)	0.7860 (7)	0.0254 (11)	
H2A	0.1352	0.2882	0.7776	0.030*	
H2B	0.2515	0.3832	0.8801	0.030*	
C1	0.4296 (11)	0.3478 (4)	0.6606 (8)	0.0227 (11)	
H1	0.4233	0.2994	0.5637	0.027*	

### Atomic displacement parameters (Å^2^)

**Table:**

	*U*^11^	*U*^22^	*U*^33^	*U*^12^	*U*^13^	*U*^23^
I1	0.0230 (3)	0.0215 (3)	0.0217 (4)	0.00012 (9)	0.0054 (2)	−0.00041 (10)
N1	0.022 (2)	0.030 (2)	0.023 (3)	−0.0030 (18)	0.0030 (19)	0.000 (2)
N2	0.021 (2)	0.022 (2)	0.034 (3)	−0.0020 (17)	0.008 (2)	0.0011 (19)
C1	0.023 (2)	0.026 (3)	0.019 (3)	0.002 (2)	0.001 (2)	0.000 (2)

### Geometric parameters (Å, º)

**Table:**

N1—C1	1.301 (7)	N2—H2A	0.8999
N1—H1A	0.9001	N2—H2B	0.9000
N1—H1B	0.9001	C1—H1	0.9500
N2—C1	1.309 (8)		
			
C1—N1—H1A	119.7	H2A—N2—H2B	120.0
C1—N1—H1B	120.3	N1—C1—N2	125.8 (5)
H1A—N1—H1B	120.0	N1—C1—H1	117.1
C1—N2—H2A	119.7	N2—C1—H1	117.1
C1—N2—H2B	120.3		

### Hydrogen-bond geometry (Å, º)

**Table:**

*D*—H···*A*	*D*—H	H···*A*	*D*···*A*	*D*—H···*A*
N1—H1*A*···I1	0.90	2.77	3.612 (5)	156
N2—H2*A*···I1^i^	0.90	2.74	3.622 (4)	166

Symmetry code: (i) *x*−1, −*y*+1/2, *z*+1/2.
